# Genetic modifiers of synucleinopathies—lessons from experimental models

**DOI:** 10.1093/oons/kvad001

**Published:** 2023-03-09

**Authors:** Rachel Min Qi Lee, Tong-Wey Koh

**Affiliations:** Temasek Life Sciences Laboratory, 1 Research Link, Singapore, 117604, Singapore; Temasek Life Sciences Laboratory, 1 Research Link, Singapore, 117604, Singapore; Department of Biological Sciences, National University of Singapore, Block S3 #05-01, 16 Science Drive 4, Singapore, 117558, Singapore

**Keywords:** Drug targets, Lewy bodies, Parkinson’s disease, SNCA, α-synuclein, Genetic modifiers

## Abstract

α-Synuclein is a pleiotropic protein underlying a group of progressive neurodegenerative diseases, including Parkinson’s disease and dementia with Lewy bodies. Together, these are known as synucleinopathies. Like all neurological diseases, understanding of disease mechanisms is hampered by the lack of access to biopsy tissues, precluding a real-time view of disease progression in the human body. This has driven researchers to devise various experimental models ranging from yeast to flies to human brain organoids, aiming to recapitulate aspects of synucleinopathies. Studies of these models have uncovered numerous genetic modifiers of α-synuclein, most of which are evolutionarily conserved. This review discusses what we have learned about disease mechanisms from these modifiers, and ways in which the study of modifiers have supported ongoing efforts to engineer disease-modifying interventions for synucleinopathies.

## INTRODUCTION

Synucleinopathies refers to a group of progressive neurodegenerative diseases that are characterized by proteinaceous inclusion bodies enriched in α-synuclein (α-syn) [1]. These include Parkinson's disease (PD), Dementia with Lewy Bodies (DLB), Alzheimer's disease (AD) and multiple system atrophy (MSA) [2–6]. In PD, DLB and subsets of AD, α-syn inclusion bodies are mostly found in neurons, while in MSA, they are found in oligodendrocytes. Clinically, PD is defined by the presence of levodopa-responsive motor symptoms like bradykinesia with either tremor or rigidity or both [7]; PD patients often live many years with the disease and develop dementia at later stages. DLB is characterized by the co-occurrence of dementia and motor symptoms within a year of the appearance of either symptom [8]. On the other hand, MSA is characterized by autonomic failure and poorly levodopa-responsive motor symptoms [9]. Individual patients with each type of synucleinopathy show considerable variations in how their symptoms manifest and progress.

α-syn is a small protein of 140 amino acids, which is encoded by the *SNCA* gene. Genome-wide association studies (GWAS) of non-familial forms of PD and DLB show strong associations of single nucleotide polymorphisms (SNPs) within or near *SNCA* with the respective diseases [10,11]. In addition, rare mutations in the *SNCA* gene have been associated with the familial forms of various synucleinopathies. Hence, missense mutations A53T, A30P, E46K, G51D and H50Q have been found in human pedigrees with PD [12–17]. In addition, rare duplications and triplications of the wildtype *SNCA* gene are also associated with familial PD [18–20], indicating that increased levels of wild type α-syn protein is sufficient to cause the disease. Interestingly, the E46K missense mutation has also been associated with DLB [21] and the G51D mutation with MSA [22]. While mutations in *SNCA* can underlie multiple forms of synucleinopathies, the distinct pathology of each disease suggests that additional genetic or environmental modifiers may influence how the pathology unfolds.

The functions of α-syn are still a matter of much debate. In the human brain, *SNCA* transcripts are found mainly in neurons and oligodendrocytes; in addition, moderate levels of transcripts are found in microglia and minute levels in astrocytes [23]. In neurons, α-syn has been localized mainly to the presynaptic compartment and neurites [24], but is also associated with mitochondria, synaptic vesicles and other subcellular compartments [25]. The normal neuronal functions of α-syn are thought to include synaptic vesicle trafficking, synaptic plasticity and mitochondrial function; the reader is referred to excellent reviews that discuss normal functions of α-syn [24–27].

**Table 1:** **Human orthologs of *SNCA* modifiers from a fly genetic screen conducted by Ren et al., (2022)** [48]

Gene	Disease Association	Molecular Function	Perturbations in PD Brains
*CDC27*	328 kb from PD GWAS SNP *rs11658976 [**10**]*	Adaptor protein for a ubiquitin ligase complex [323]	mRNA decreased in PD brains [65]
*ABCB7*	X-linked sideroblastic anemia with ataxia [323]	Mitochondrial transporter for Fe-S cluster [49]	Protein upregulated in MPTP mouse model [68]
*CDAN1*	1 kb from bipolar type 1 GWAS SNP *rs112968809* [324]	Role in nuclear envelope integrity [323]	No data
*MTA1, 2, 3*	*MTA1*: CNV associated with inflammatory bowel disease [325] MTA3: Insomnia GWAS SNP *rs6734957* in intron [269]	Transcriptional co-activator/repressor [323]	*MTA1* mRNA and MTA1 protein decreased in PD brains [66]
*MED13, MED13L*	*MED13* is 103 kb from PD GWAS SNP *rs61169879 [**10**]*	Subunit of Mediator kinase module [326]	Protein upregulated in fly and mouse *SNCA* models [48]
*NGRN*		Implicated in mitochondrial translation [51]	No data
*HECA*	Common variants close to genome-wide significance in association with coronary artery disease in diabetes [327]; rare variants associated with congenital heart disease [328]	Regulates insulin signaling in flies [128]	No data
*GARS*	Charcot–Marie–Tooth Disease Type 2D and Distal Hereditary Motor Neuronopathy Type VA [323]	Glycyl-tRNA synthetase 1 [323]	mRNA decreased in PD brains [64,65]
*COX20*	Mitochondrial complex IV deficiency nuclear type 11 [323]	Cytochrome c oxidase assembly factor [323]	No data
*RBPMS, RBPMS2*	*RBPMS2* is 8 kb from coronary artery disease GWAS SNP *rs6494488 [**329**]*	RNA binding protein [323]	No data
*NDUFA9*	Mitochondrial complex I deficiency, nuclear type 26 [323]	Mitochondrial complex I component [323]	mRNA decreased in PD brains [64,65]
*NR1D1, NR1D2*	*NR1D1* intron contains multiple sclerosis GWAS SNP *rs883871* [330]	Homolog of nuclear receptor implicated in circadian rhythm regulation [267]	*NR1D1* mRNA and NR1D1 protein decreased in rat brains treated with LPS and rotenone [331]

α-syn is known to form pathological aggregates in neuronal cell bodies and neurites, known as Lewy bodies and Lewy neurites, respectively [28]. This is favored by its high abundance at synapses, where its concentration (~ 22 μM) is similar to those of clathrin heavy chain and SV2A [29]. Interestingly, genetic evidence suggests that further increases in the concentration of α-syn are a driving force for neuronal toxicity [18,30–32]. *In vivo* factors that are thought to drive the misfolding of α-syn into pathological aggregates are: (1) total α-syn levels, (2) mitochondria impairment and (3) membrane associations, as summarized in a recent review [33]. *In vitro* studies have demonstrated that high concentrations of α-syn can progressively form aggregates such as the smaller soluble oligomers and the higher-order insoluble fibrils [34]; such fibrils are thought to be the precursors of Lewy bodies [35]. Meanwhile, the mechanisms of α-syn neurotoxicity is currently debated; while the prevailing view is that both oligomers and fibrils contribute to toxicity [36], an alternative view suggests that excessive membrane binding by monomeric α-syn also contributes to pathology [37]. Besides causing pathologies within a given cell, α-syn aggregates are known to spread from one cell to another to seed further aggregations and spread the pathology across brain regions [33,38].

The occurrence of α-syn spreading in experimental models is consistent with Braak's hypothesis which states that for at least a subset of PD cases, Lewy pathology spreads from the olfactory system or the enteric nervous system to the central nervous system [28]; this hypothesis is based on the analysis of Lewy pathology in postmortem brains from patients at various stages of PD [39]. Subsequent work has resulted in the view that Lewy pathology in the periphery and brainstem may be responsible for prodromal symptoms such as rapid eye movement (REM) sleep behavior disorder, constipation and anosmia that may emerge before the diagnosis of motor symptoms in PD, in a decades-long process of disease progression [40–42].

As summarized above, genetic, biochemical and pathological evidence point to key roles for α-syn in the pathogenesis of synucleinopathies. However, currently known GWAS variants account for only up to a third of the genetic risk of PD [10]. One way to expand our understanding is through the characterization of genetic modifiers of *SNCA* that alter the risk of synucleinopathies [43,44]. While GWASes of genetic modifiers of *LRRK2* and *GBA* with respect to PD have been performed in human populations [45,46], there is no similar human study for *SNCA*, which shows the strongest association with PD. However, studies done using model organisms and cell lines to discover *SNCA* modifiers have revealed much about potential mechanisms of synucleinopathies; the current review will discuss these findings and their implications for novel therapeutic interventions.

## GENETIC MODIFIERS OF *SNCA*

Genetic modifiers are genes that modify a clinical phenotype of a disease, i.e. the measure of the clinical variability in the population of affected individuals [47]. In the case of synucleinopathies, this may simply be a measure of *SNCA*-associated neurodegeneration or α-syn aggregation. The search for these modifiers can help elucidate genetic interactions of *SNCA*, hence shedding light on pathways important in causing disease in synucleinopathies. Once validated, these target proteins and pathways may then be exploited to develop therapeutics.

### A *Drosophila* screen for *SNCA* modifiers

In our recent work, we described a screen in flies that identified twelve genetic modifiers in a model of *SNCA*-associated neurodegeneration (Table 1) [48]. Three-quarters of the modifiers have known roles in metabolism. *ABCB7*, *CG4553/NGRN*, *GlyRS/GARS1*, *COX20* and *ND-39/NDUFA9* encode mitochondrial proteins [49–55], while knockdown of the *Drosophila* nuclear protein MTA1-like led to mitochondrial defects [56]. On the other hand, *MED13* and *CDC27* encode subunits of protein complexes that regulate different aspects of glycolysis [57–59]. Lastly, the human orthologs of *Eip75b*, *NR1D1* and *NR1D2* regulate many aspects of energy metabolism [60,61]. The large proportion of modifiers with metabolic functions underscores the importance of energy metabolism in synucleinopathies, as observed previously [62,63].

Since all 12 modifiers were loss-of-function mutations that enhance *SNCA*-associated neurodegeneration, we propose that subsets of their mammalian orthologs show expression changes in two plausible scenarios (Table 1): (1) a constitutively expressed neuroprotective gene that is downregulated through a pathological mechanism leading to enhanced neurodegeneration; (2) a neuroprotective gene that is induced upon neuronal stress to compensate for the primary pathological event. Genes that fit Scenario 1 are *CDC27*, *GARS1*, *NDUFA9* and *MTA1*. Transcripts of *CDC27*, *GARS1* and *NDUFA9* were found to be significantly downregulated in meta-analyses of transcriptomes of PD brains [64,65]. Similarly, *MTA1* mRNA and its associated protein are downregulated in PD substantia nigra samples [66]. In addition, MTA1 is a coactivator that drives transcription of the *TH* gene [67], which is essential for dopaminergic function. Genes that fit Scenario 2 are *ABCB7* and *MED13*. *ABCB7* was upregulated in a 1-methyl-4-phenyl-1,2,3,6-tetrahydropyridine (MPTP) mouse model of PD [68], suggesting that its anti-apoptotic function may be induced by neuronal stress [69]. In our work, *MED13* was induced in flies and mice with elevated levels of *SNCA* expression and was significantly associated with PD in eQTL analysis and TWAS [10,70,71]. Hence, the mammalian orthologs of six *SNCA* modifiers show perturbations in expression levels in PD brains and, in some cases, the relevant animal models.

### *SNCA* modifiers from other experimental models

Since the discovery of the central role of *SNCA* in both familial PD and sporadic PD [2,12,72], there have been many efforts to identify genetic modifiers of this gene. Our work summarized above is just the latest in the field. An overview reveals that a number of pathways are important in causing disease in synucleinopathies. In this review, we have compiled a list of genetic modifiers of *SNCA*, classified into two types (Table 2). A gene is a *Type 1 Modifier* when its function is inversely correlated with the level of α-syn pathology; a *Type 1 Modifier* has a neuroprotective function. On the other hand, a *Type 2 Modifier* has functions that are directly correlated with the level of α-syn pathology; inhibiting a *Type 2 Modifier* is likely to protect neurons. For the sake of consistency, we will use the name of the human ortholog to refer to each modifier where feasible.

**Table 2:** Genetic Modifiers of *SNCA*

Pathway	Modifier Type	Genes
Mitochondria	1	Protein transport: *TOMM20* [104]; *TIMM9 [**332**]*; *CSNK2B [**333**]* Mitochondrial protein synthesis: *PPARGC1A* (PGC-1α) [101–103]; *REST* [103]; *NGRN*, *GARS1* [48] Mitochondrial chaperone: *TRAP1* [105] Mitochondrial kinase: *PINK1* [334–336] Fusion: *OPA1* [87]; *PINK1*, *PARK7* (DJ-1), *PRKN* (Parkin) [337] Fission: *DNM1L* (Drp1) [83]; Fragmentation: *PAPSS1*, *PAPSS2*, *PTPN23* [333] Mitophagy: *CNTNAP4* [338]; *TMEM175* [339]; *FBXO5* [340]; *CDC37 [**333**]* Electron transport chain: *COX20*, *NDUFA9* [48] Calcium: *CAMK2D*, *ITPKB* [341] ER-mitochondria calcium transport: *ITPKB* [252] ROS: *NCEH1*, *SOAT1* [191]; GSH: *MED13* [48] NAD^+^: *FBXO5* [340] Iron–sulfur cluster transport: *ABCB7* [48]
	2	Mitochondrial function: *CHCHD2/10* [342] Fusion: *PDE4B* [343] Fission: *DNM1L* (Drp1) [86] Mitophagy: *SIRT1* [250] Calcium: *TLK2* [253]; *MICU3* [341]
Glycolysis	1	*GPI* [48,106]; *MED13*, *PFKM*, *HIF1A* [48]; *GAPDH* [106]
	2	*LDHA* [48]; *AMPK* [344];
Antioxidant/glutathione metabolism	1	Oxidative stress: *PARK7* (DJ-1) *[**114**,**332**]*; *UBE2I* (Ubc9) [345]; *FOXO3* [346]; *NFE2L2* (Nrf2) [112,113,347,348] [112,113], *MAFK* [113]; *PPARGC1A* (PGC-1α) [102]; *TXN [**332**]*; *CTH*, *GCLM*, *HPGDS* [116] Nitrosative stress: *COX4I1/2* [349]; *KLF11*, *KLF15* [350] Lipid peroxidation: *RSPA* [136] Detoxification: *CYP2B6* [249]
	2	Oxidative stress: *PREP* [351]; *KEAP1* [113] Nitrosative stress: *COX4I2* [349]
Insulin-like signalling (IIS) pathway	1	*AMPK* [352]; *IGF1*, *AKT1* [120]; *FOXO*, *TFPI* [106]; *KDM1A* [136]; *RPS6* [353]; *IDE* [354]; *HECA* [48]; *MLST8* [350]
	2	*IGF1R*, *IRS1* [106]; *MTOR* [355]
Cytoskeleton	1	*SPTAN1* (Spectrin) [83]; *LRRK2* [237]; *PFDN5* (Prefoldin), TBCA [356]; *TTC7A*, *TTC7B* [357]; *CFL1* [135], *GSN* [83,135]; *ACTG1* (Actin) [136]; *HDAC6* [358]; *ITGA8* [340]
	2	*ACTB* (Actin) [83,359] [83]; *FHOD1*, *FHOD3* [83]; *MAPT* (Tau) *[**83**]*; *TPPP* [360]; *SIRT2* [245]; *LRRK2* [237]
α-syn accumulation (transcription)	1	*ZSCAN21* [146]; *CEBPD* [141]; *TRIM41* [144]
	2	*ZSCAN21* [144–146]; *GATA2* [142]; *TP53* (p53) [143]; *TRIM17* [144]; *HMOX1* [361]; *CEBPB* [362]
α-syn accumulation (Degradation pathway unknown)	1	Target for degradation: *STUB1* (CHIP) [363,364]; *USP8*, *USP13* [365] ER stress: *GBA1* [366]; *SIRT1* [249]; *COX4I1/2* [349]; *MANF* [367]; *PISD* [368] Protein misfolding: *VPS35* [369]
	2	Ubiquitination: *BAG5*, *HSPA1A* (Hsp70) [364]; *USP9X* [365] ER stress: *CASP12* [370]; *COX4I2* [349]
α-syn accumulation (autophagy)	1	*AMPK* [352]; *ATG5* [87,371]; *VPS41* [372,373]; *STUB1* (CHIP) [374]; *BECN1* [375]; *MANF* [367]; *NFE2L2* (Nrf2) [376]; *PFDN2*, *PFDN5* [377]; *PINK1* [378]; *SQSTM1* (p62) [371]; *SIRT1* [243]; *TFEB* [177,243,379]; *TMEM175* [339]; *TERT* [380]; *TTC7A*, *TTC7B* [357]; *HSPA1B* (Hsp70) [381]; *CREBRF* [253]; *SNCAIP* (Synphilin-1) [152,154]; *SCARB2* (LIMP-2) [382]; *VPS35* [383]; *SMPD1* [197]; *ATP13A2* (*PARK9*) [384–387]; *PLD1* [388]; *NSF* [341,389]; *RAB27B* [390]; *RAB7A* [391]; *RAC1* [392]; *PSAP* [393]; *CTSD* [394,395]; *HMGB1* [375,396]; *FOXO3* [346]; *GBA1* [175,176,199,366,397,398]; *CFL1*, *GSN* [135]; *LRRK2* [314,399,400]; *CNTNAP4* [338]; *NSFL1C* [401]; *GBA1* [402]; *HDAC6* [358]; *TAX1BP1* [340]; *VPS41*, *GIPC1*, *ATG7* [403]; *BAG3* [404]; *RAB1A* [405] Ubiquitination: *NEDD4* [168,406] Deubiquitination: *USP9X* [173] SUMOylation: *UBE2I* (Ubc9) [345]
	2	*LPCAT1* [407]; *NLRP3* [408]; *UCHL1* [409]; *PREP* [351]; *DCLK1* [410]; *PARP1* [243]; *SIRT1* [250]; *MTOR* [355]; *BAG5* [411]; *LGALS3BP*, *LOXL1* [412]; *ATG5* [404]; *TLK2* [253] Deacetylation: *SIRT2* [246] Deubiquitinaition: *USP8* [413] Calcium: *GSK3B* [414]
α-syn accumulation (proteasomal degradation)	1	*STUB1* (CHIP) [374]; *NFE2L2* (Nrf2) [415]; *RER1* [416]; *ATP13A2* (*PARK9*) [386]; *PINK1* [336]; *PRKN* (Parkin) [417,418]; *CTSD* [395]; *PSMA6* [353]; *NSFL1C* [401]; *PPP6C* [401]; *USP9X* [173] Protease: *ECE1* [419]; *USP10* [308]; *ADAMTS19* [340]; *HTRA2* (Omi) [178]
	2	*UCHL1* [420]; *PREP* [351]; *PIAS2* [421]; *USP13* [422] Protease: *CAPN1* (Calpain) [423]

(Continued)

**Table 2:** Continued

Pathway	Modifier Type	Genes
Chaperone	1	*ARSA* [424]; *HSPA1A* (Hsp70) [147,425–432]; *HSPA8* (Hsc70) [147,433]; *DNAJB6* [425]; *DNAJB1*, *HSPA4* (Hsp110 family), [433]; *DNAJA1* (Hsp40), *TOR1A* (TorsinA) [426]; *RPS3A* [434]; *CRYAB* (HspB5; αB-c), *HSPB1* (Hsp27) [435,436]; *IDE* [354]; *HSP90AA1* (Hsp90) [437]; *HSPA4L* (Hsp110) [438]; *CLPB* (Hsp104) [439]; *DNAJB1* (Hsp40), *PARK7* (DJ-1) *[**157**,**332**]*; *PSMA6* [353]; *YWHAH* (14–3-3η) [440]; *YWHAQ* (14–3-3θ) [441,442]; *CACYBP* (SIP) [443]; *PCSK1N* (proSAAS) [444] Extracellular: *HSPA1A* (Hsp70) [445] Upstream: *HSF1* [446]; *SIRT1* [447]; *HDAC6 [**448**]*; *PPP1R3C* [350]; *KDELR2* [412]
Direct interaction	1	*SNCB* (β-syn) [449–453]; *SNCAIP* (Synphilin-1) [152,153,155]; *HDAC6* [454]
	2	*TARDBP* (TDP-43) [455,456]; *DDX10* [457]; *DUSP11* [412]; *TPPP* [360]; *MAPT* (Tau) [458–460]; *TGM2* (tTGase; TG2) [461]; *SNCAIP* (Synphilin-1) [156,462–464]; *PREP* [465]; *PARP1* [466]
Post-translational modifications	1	Ubiquitination: *NEDD4* [467]; *ATP13A2* (*PARK9*) [386]; *SYVN1* [308] Phosphorylation: *CDK4*, *CDK6*, *STK3* [187]; *PTPA* [401]; *SEPTIN4* [468]; *PRKN* (Parkin) [469]; *CSNK1G2* (CKI) [470] SUMOylation: *CBX4* (Pc2) [471]
	2	Ubiquitination: *TRAF6* [472]; *SIAH1* [171,172]; *SIAH2* [171] Phosphorylation: *GRK3*, *GRK5* [249] Y39: *ABL1* [149]; Y125: *FYN* [473,474]; *SRC* [474] S129: *CSNK1G2* (CKI) [188,475]; *CSNK2A2* (CK2) [475–478]; *GRK2* [479]; *GRK5* [479,480]; *LRRK2* [481]; *PLK1* [482]; *PLK2* [482–484]; *PLK3* [484]; *SGK2* [188] S87: *DYRK1A* [485] N-terminal acetylation: *NAA20* (*NAT5*) [188] Not phosphorylate: *CDK14* [486]
Vesicular/membrane trafficking	1	ER-Golgi: *YKT6* [350,487]; *RAB1A* [184–186,350,405]; *RAB3A*, *RAB8A* [186]; *CHMP2B* [106]; *PPP6C* [401]; *YIF1A* [358]; *SEC22B* [403]; *VPS35* (*PARK17*) [342] Endocytic trafficking: *RAB11A*, *RAB13* [488]; *RAB29* (*PARK16*/*RAB7L1*), *SYNJ1* (*PARK20*) [342]; *ALS2 [**332**]*; *NSF* [341]; *GIPC1* [403] Endocytosis: *AP2A2* (AP-2), *EPS8L2*, *RAB7A* [187]; *AAK1* [358]; *GAK* [489]; *SORL1* [342] Golgi-endosome: *GGA1 [**332**]*; Golgi clearance and turnover: *GAK*, *DNAJC6* [490]
	2	Endocytosis: *BIN3*, *CLTC [**188**]*; *CHMP4B* [412] Endosome maturation: *VPS33A* [188]
Lipid metabolism	1	Phospholipid: *CHKB* [136]; *SYNJ1* (*PARK20*) [342]; *INPP5F* [341] Sterol metabolism: *NCEH1*, *SOAT1* [191]; *AMFR*, *GPI* [106]; *LIPA*, *NPC1* [190]; *OSBP*, *OSBPL7* [192,350]; *SIGMAR1* [188]; *SYVN1* [350] Very long chain fatty acid synthesis: *ELOVL1*/*4*/*6*/*7* [203] Sphingolipid/ceramide: *PLA2G6* (*PARK14*) [196,201]; *B4GALNT1 [**200**]*; *GBA1* [190,199]; *SCARB2*, *SMPD1* [190]; *CERS2* [249] Glycosaminoglycan: *GLB1*, IDUA, MAN2B1, MANBA [190]
	2	Phospholipid: *LPCAT1* [407] Sterol metabolism: *OSBP*, *OSBPL7* [192]; Long-chain fatty acid: *ACSBG1* [412] Mevalonate-ergosterol: *DOLPP1*, *RABGGTA* [350]
α-syn spread	1	*YWHAQ* (14–3-3θ) [442]; *ATP13A2* (*PARK9*), *LRRK1*, *PARK7* (DJ-1), *PRKN* (Parkin), *VPS35* [214]; *HSPA4L* (Hsp110) [438]; *VCP* [491]; *RAB8B*, *RAB13*, *SYTL5* [488]; *CTSD* [394]; *GBA1* [398]; *ADAMTS19*, *FBXO5*, *ITGA8*, *SLC30A3*, *TAX1BP1* [340]; *CHRNA7* [492] Uptake: *CDNF* [493] Secretion: *GABBR1* [223]; *ARSA* [424]; *GBA1* [198,494]; *ATP13A2* (*PARK9*) [225,386]; *HDAC6* [224]; *RAB11A* [495,496]; *RAB27B* [390]
	2	*APOE4* [497,498]; *LPCAT1* [407]; *LRRK2* [215]; *PARP1* [466]; *PREP* [351]; *NFX1*, *POLR2A* [340]; *PRNP* [499] Uptake: *LRP1* [500]; *LAG3* [216]; *SLC35B2*, *MYO7B* [501]; *RAB5A* [502] Secretion: *ABCC8* (SUR1) [223]; *PIAS2* [421]; *MAOB* [503]; *TPPP* (p25α), *RAB1A*, *RAB7A*, *RAB8A*, *ATG5* [224]; *PIKFYVE* [213]
Synaptic impairment	1	*RAB11A* [504]; *TOR1A* [505] Dopaminergic neurotransmission: *SEPTIN4* [468]; *PRKN* (Parkin) [506]
	2	*SLC6A2* (DAT-1) [505]. *SYN3* [507]; *ADORA2A* [508]; *PRNP* [509]; *FYN* [473,474]; *SRC* [474] Dopaminergic neurotransmission: *PREP* [351]; *TNK2* (*ACK1*) [510]
Neuroinflammatory responses	1	*NR4A2* (Nurr1) [232]; *PRKN* (Parkin) [469]; *NR1D1* [48]
	2	*LRRK2* [215,239,511] [215,511]; *NLRP3* [408]; *TGM2* (tTGase; TG2) [231]
Calcium homeostasis	1	*PPP3CA*, *PPP3CB*, *PPP3R1* [401]; *CRTC2*, *PPP3CC* [254]
	2	*NFATC4*, *PPP3CC* [254]; *FKBP1A* (FKBP12) [512]
Cell cycle	1	*PLK2* [308]; *PPP6C* [401]; *PPP2R2B* (*ATXN12*) [342]; *CDC27*, *HECA* [48]; *RNR1* [513]
Circadian rhythm	1	*MTA1/2/3*, *NR1D1/2* [48]

(Continued)

**Table 2:** Continued

Pathway	Modifier Type	Genes
Other pathways	1	Translation: *ATXN2*, *EIF4G1* (*PARK18*) [342]; *RPSA* [136]; *RPS6* [353]; *PABPC1* [342]; *GARS1* [48]; *RSPA* [136] Transcription: *ATXN7* [342]; *MTA1/2/3, NR1D1/2*, *RBPMS*, *RBPMS2* [48]; *EXOSC9* [333] Apoptotic pathway: *HSPB1* (HSP27) [514]; *SNCAIP* (Synphilin-1) [152]; *ATP13A2* (*PARK9*) [387] α-syn sequestration on intracellular membranes: *GDI1*, *RAB3A* [515] Ion homeostasis: *DRD4*, *NPY1R* [358]; *ATP13A2* (*PARK9*) [342] Potassium homeostasis: *PPP6C*, *PPP6R1*, *PPP6R2*, *PPP6R3* [401] Zinc homeostasis: *ADAMTS19*, *SLC30A3* [340] Iron: *ABCB7* [48] Mn^2+^: *ATP13A2* (*PARK9*) [308] Acetylcholine signaling: *ACTG1*, *DPYSL2* (CRMP2), *KDM1A* [136] Phosphodiesterase: *PDE9A*, *PDE8B* [308] Neurotrophic factor signalling: *NR4A2* (Nurr1) [516,517]; *RET* [517]
	2	Translation: *TARDBP* (TDP-43) [455]; *EIF4G1* (*PARK18*) [369]; *POLR2A* [340] Apoptotic pathway: *PDE4B* [343]; *CASP3*, *CASP9*, *CASP12* [370]; *FOXO3* [346] Parthanatos: *PARP1* [466] α-syn sequestration on intracellular membranes: *Hsp90AA1* (Hsp90) [515] Polyamine pathway: *SLC22A2* [518]
Not mentioned in text/No clear category	1	*RTCB* [403]; *SUGT1* (upregulate *PINK1* and *PARK9*) [519]; *ATP13A2* [403]; *GBA1* [520]; *GAK*, *DNAJC6*, *RIT2*, *VPS13C* [399,521]; *PRKN* (Parkin) [522,523]; *TRIM28* [524]; *DNAJC5* [190]; *RAB39B* [525]
	2	*PRKN* (Parkin) [526]; *MAPT* (Tau) [527]; *PARK7* (DJ-1) *[**528**]*; *TARDBP* (TDP-43) [529]; *SIDT1*, *SIDT2* [510]; *DCLK1* [412]; *LRRK2*, *RAB29* [341]; *SIN3A* [530]; *SERF1A*, *SERF2* [531]

The human orthologs of genetic modifiers of *SNCA* identified in various experimental models are listed by the pathways they are involved in. We have classified the modifiers into two types. A gene is a *Type 1 Modifier* when its function is inversely correlated with the level of α-syn pathology; such a gene can be inferred to possess neuroprotective function. On the other hand, a gene is a *Type 2 Modifier* when its function is directly correlated with the level of α-syn pathology; such a gene is a candidate target for pharmacological inhibitors for neuroprotection. In brackets are alternative protein and gene names used in literature.

#### Mitochondria functions

Mitochondria are powerhouses of the cell, while also important in cell development, cell cycle and cell death [73]. The important role of mitochondria in synucleinopathies is highlighted by the reduction of mitochondria copy number detected in postmortem PD brains [74]. This is consistent with key genes underlying familial PD, such as *PINK1* and *PRKN/parkin,* which are important for mitochondria homeostasis [75]. In addition, α-syn has been shown to disrupt mitochondria functions via multiple mechanisms [76–79]. Accordingly, many *SNCA* modifiers identified in experimental models are important for mitochondrial function.

Mitochondrial fusion and fission events are dysregulated in synucleinopathies, correlating with impaired mitochondrial bioenergetics. Mitochondrial fission can lead to either proliferation or degradation of damaged components via mitophagy, depending on the physiological context and the molecular machinery that is recruited for the fission event [80]. Conversely, fusion can promote content exchange and maintenance of mitochondrial DNA [81]. The dynamic balance between the two opposing processes is probably more important than the level of either process alone. For example, mice deficient in fission through disruption of *Mff*, or those deficient in fusion through disruption of *Mfn1*, suffer early lethality, but double mutants are largely normal [82]. Various experimental models expressing α-syn have shown alterations of mitochondrial morphology in the directions of either excessive fission or fusion. For example, a fly model with a high level of wild type human α-syn expression and a mouse model expressing an A53T mutant version of human α-syn both shows increased volumes of mitochondria residing at neuronal cell bodies in the respective brains [83,84]. In contrast, a different fly model expressing wild type human α-syn at a relatively low level, primary cultured neurons from A53T α-syn transgenic mice and several human cell line α-syn transfection models show mitochondrial fragmentation in either axons or cell bodies [76,85–88]. While many factors can potentially account for the contrasting effects of α-syn on mitochondrial morphology, a plausible factor could be the different levels of α-syn expression. Hence, increased mitochondrial volumes are associated with relatively higher levels of α-syn (in both flies and mice), while decreased mitochondrial volumes are associated with relatively low α-syn levels; transfected cell lines and primary neuronal cultures may express α-syn at levels lower than that found in mature neurons [29].

Just as there are contrasting changes to mitochondrial morphology in response to α-syn expression, a key fission protein, dynamin-related protein 1 (Drp1), encoded by *DNM1L*, has been found to modify α-syn toxicity in opposite directions. In the fly model expressing high levels of α-syn and showing enlarged mitochondria, co-overexpression of *DNM1L* partially suppressed neuronal loss, mitochondrial reactive oxygen species (ROS) and locomotor decline [83]. In the fly model expressing relatively lower of α-syn and showing mitochondrial fragmentation, reduction of *DNM1L* function using the drug, Mdivi-1, ameliorated mitochondrial aging, as measured by the MitoTimer transgene [85,89]. Similarly, in SH-SY5Y cell lines transfected with α-syn that show mitochondrial fragmentation, inhibiting fission via *DNM1L* knockdown or promoting fusion through *OPA1* overexpression suppressed cell death and ROS levels [86,87]. Currently, it is unclear whether the contrasting effects of α-syn expression on mitochondrial morphology and the opposing effects of *DNM1L* on α-syn toxicity result from experimental artifacts or these different results reflect different manifestations of α-syn toxicity in different neuron types or stages of disease. Careful examination of Drp1 levels in various postmortem brain regions of PD patients at different stages of the disease may help resolve this issue.

Mitochondrial fragmentation is known to facilitate mitophagy [90], and evidence shows that α-syn may be associated with reduced mitophagy. Miro1, encoded by *RHOT1*, is an outer mitochondrial membrane protein important in mitochondrial transport through its interactions with the microtubule motor kinesin [91–93]. For mitophagy to occur, Miro1 must be removed from damaged mitochondrial surfaces to arrest mitochondrial motility [94,95]. This process seems to be disrupted in Parkinson’s disease, as α-syn and Miro1 were both found to be upregulated in PD postmortem brains [96]. Conversely, Miro1 knockdown in iPSC-derived neurons expressing α-syn increased mitophagy as measured by the fusion of mitochondria-containing autophagosomes with acidic lysosomes [96]. Indeed, reducing Miro1 suppresses dopaminergic neurodegeneration in flies expressing α-syn [96,97] and reduces α-syn toxicity in yeast [98]. A small molecule that promotes Miro1 degradation has been identified, which rescues the mitochondrial motility and clearance of defective mitochondria in iPSC-derived neurons [97], indicating the potential of targeting Miro1 as a therapeutic for synucleinopathology.

**Figure 1:**
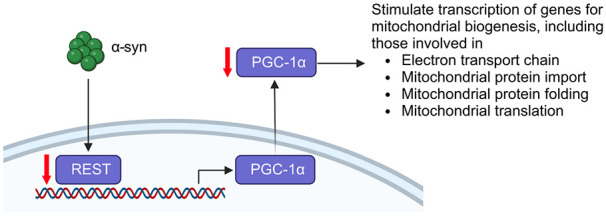
Pathways by which genes important for mitochondrial biogenesis that modify α-syn pathology. PGC-1α, encoded by *PPARGC1A*, can stimulate transcription of genes for mitochondrial biogenesis. These genes are downregulated in PD brains. *REST* induces PGC-1α expression and is downregulated in iPSC-derived dopaminergic neurons carrying the *SNCA* triplication. Created with BioRender.com

Furthermore, mitochondrial biogenesis is also important to maintain an adequate and functional mitochondrial population (Figure 1). PGC-1α, encoded by *PPARGC1A*, and a master regulator of mitochondrial biogenesis and ROS scavenging [99]. Using a systems biology approach, PGC-1α transcriptional targets were found to be under-expressed in PD brains [100]; it was demonstrated to be an *SNCA* modifier when its overexpression suppressed *SNCA*-associated death of cultured dopaminergic neurons [100]. Indeed, overexpressing PGC-1α also rescues α-syn toxicity in mice and a zebrafish model exhibiting mitochondrial pathology [101,102]. Interestingly, *REST*, which induces PGC-1α expression, was downregulated in iPSC-derived dopaminergic neurons carrying the *SNCA* triplication, while its overexpression reduced cell death in response to MPTP-induced dysfunction of mitochondrial complex I in SH-SY5Y cells [103]. Hence, the evidence suggests that upregulation of mitochondrial biogenesis may be neuroprotective.

In addition, other *SNCA* modifiers are involved in other aspects of mitochondria function, such as *COX20* and *NDUFA9* (electron transport chain) [48], *TOMM20* (protein transport) [104], *GARS* and *NGRN* (mitochondrial protein synthesis) [48] and *TRAP1* (mitochondrial chaperone) [105]. Evidently, the association between α-syn and mitochondrial dysfunction is significant and reducing this dysfunction can play a major role in treatment of synucleinopathies.

#### Glycolysis and glutathione synthesis

A recent review summarizes various studies indicating that elevation of glucose metabolism is neuroprotective in neurodegenerative diseases [63]. In flies overexpressing *SNCA*, overexpression of either of the two glycolytic enzymes, *GPI* and *PFK*, partially rescued neurodegeneration [48]. Conversely, knockdown of *GPI* worsened neurodegeneration in worms and flies overexpressing *SNCA* and led to accumulation of α-syn protein in cultured mouse neurons [106]. Interestingly, terazosin, a hypertension medication that is secondarily found to activate another glycolytic enzyme, phosphoglycerate kinase (*PGK*), is now considered a potential drug for PD [107,108]; here terazosin was found to alleviate α-syn accumulation and neurotoxicity in a few PD models [107].

**Figure 2:**
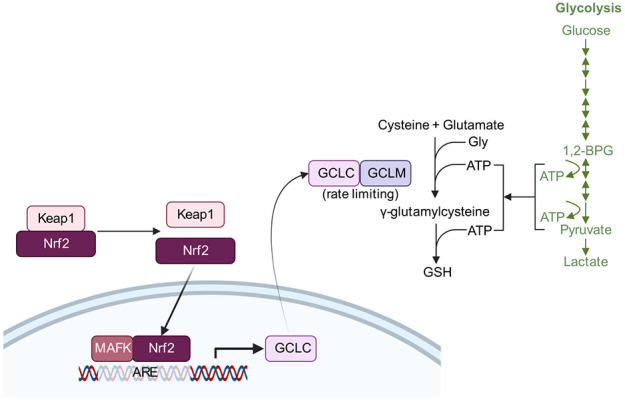
Pathways by which genetic modifiers of α-syn pathology may act. Nrf2, encoded by *NFE2L2*, can interact with MAFK and bind to the antioxidant responsive element (ARE) to drive transcription of *GCLC*, encoding a subunit of GCL (glutamate cysteine ligase). GCL catalyzes the rate-limiting step in glutathione (GSH) biosynthesis. Keap1 inhibits Nrf2. ATP required for the *de novo* synthesis of GSH is provided for by glycolysis (green). Created with BioRender.com

How is glycolysis involved in synucleinopathies? A recent study revealed that disruption of mitochondrial complex I in mice leads to parkinsonian phenotypes and upregulation of glycolysis [109]. Given that high levels of α-syn impair mitochondria functions, we examined fly and mouse models of synucleinopathy. Indeed, *SNCA* overexpression in flies and mice leads to upregulation of a subset of glycolytic enzymes [48]. While glycolysis generates metabolites for many cellular functions, one notable process is *de novo* glutathione synthesis, which involves two ATP-consuming steps [110] (Figure 2). Interestingly, metabolomics and isotope tracing by Patten et al. showed that in cells with impaired mitochondria, *de novo* glutathione synthesis increases in a manner that is dependent on the ability of the cell to upregulate glycolysis [111]. We thus examined the ratio of oxidized to reduced glutathione (GSSG:GSH) in fly neurons and found that neurons co-overexpressing *SNCA* and *GPI* showed lower GSSG:GSH ratio than those overexpressing only *SNCA* [48]; hence, these neurons likely channeled ATP from glycolysis towards GSH synthesis.

Besides glycolysis, antioxidant pathways have also been shown to be an adaptive response toward synucleinopathies. *NFE2L2* encoding Nrf2, a transcription factor regulating cellular antioxidant responses, and genes in its pathway have been demonstrated to modify α-syn-associated neurodegeneration in *Drosophila*, by increasing transcription of the catalytic subunit of GCL [112,113] (Figure 2). *PARK7* apparently also confers its protection against α-syn-mediated toxicity by increasing GCL expression [114]. Phase II detoxification enzymes involved in glutathione metabolism were also identified as enhancers of α-syn-associated toxicity in a yeast screen [115], with further validation in the fly, including the fly homolog of *GCLM* encoding the modifier subunit of GCL [116]. Hence, glutathione biosynthesis pathways are implicated as modifiers of α-syn, indicating the role of oxidative stress in α-syn pathophysiology.

#### Insulin, insulin-like growth factors and mTOR signaling

**Figure 3:**
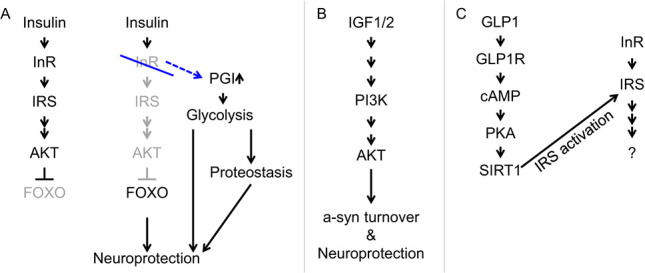
Genetic modifiers of α-syn pathology from the insulin-like signaling pathway and glycolysis pathways. (A) Disruption (blue stroke) of insulin receptor (InR encoded by *daf-2*) in worms leads to derepression of FOXO and upregulation of PGI, both of which contribute to neuroprotection; PGI upregulation in *daf-2* mutants occurs via an indirect mechanism that does not involve FOXO [106,117]. (B) Application of IGFs in mammalian cells or rodent models is neuroprotective. (C) GLP1 can potentially enhance insulin sensitivity via SIRT1 and IRS [118,119].

The insulin signaling pathway has been found to confer protection against α-syn toxicity in some experimental models. Using a human SH-SY5Y neuroblastoma cell line overexpressing *SNCA*, Kao showed that insulin-like growth factor 1 (IGF1) application rescues α-syn toxicity and aggregation [120]. Similarly, a different study using 6-hydroxydopamine (6-OHDA) treatment of SH-SY5Y cells and mouse to model PD showed that insulin-like growth factor 2 (IGF2) rescues α-syn toxicity and aggregation [121]. Both studies demonstrated that the rescue of synucleinopathy by IGFs requires a functional PI3K/AKT pathway. IGF1 is thought to rescue neurotoxicity by activating mTOR and preventing excessive autophagy in an MPTP mouse PD model [122]. Besides blocking neuronal apoptosis at the level of neuronal cell bodies, activation of AKT/mTOR signaling has also been shown to suppress axon degeneration by inhibiting macroautophagy [123]. Together these studies suggest that activating PI3K/AKT/mTOR signaling may be neuroprotective (Figure 3B).

**Figure 4:**
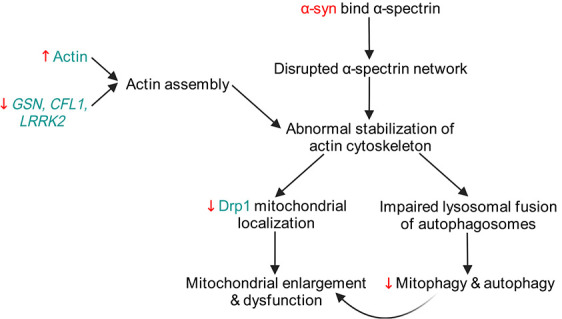
Pathways by which genetic modifiers of α-syn pathology may lead to cytoskeletal dysregulation, mainly by the abnormal stabilization of the actin cytoskeleton, resulting in altered mitochondrial dynamics and mitochondrial dysfunction. Created with BioRender.com

Interestingly, glucagon-like peptide-1 (GLP1), a peptide hormone that enhances insulin sensitivity [118,124,125] (Figure 3C), is associated with neuroprotective activities [126]. A brain-penetrant GLP1 analog is shown to protect against dopaminergic neuronal death in an α-syn preformed fibril mouse model of PD [127]; in this study, the GLP1 analog prevents microglia from converting astrocytes to a neurotoxic phenotype, hence preventing the death of dopaminergic neurons.

While the above studies suggest that activation of the insulin signaling pathway may protect neurons, others below suggest the opposite is true, at least in the following contexts. In worms and flies, disruption of insulin receptor and the insulin receptor substrate homologs alleviates dopaminergic neuron death [106] (Figure 3A). Similarly, in the *SNCA* modifier screen performed in our laboratory [48], heterozygous loss-of-function mutations in *headcase* (*hdc*)/*HECA*, a negative regulator of insulin/mTOR signaling [128,129], was found to enhance α-syn-associated neurodegeneration. In contrast to findings discussed in the previous two paragraphs, this suggests that reducing insulin/mTOR protects neurons against α-syn toxicity. It is interesting that the findings from Knight et al. and Ren et al. are based on chronic disruption of insulin/mTOR signaling, while those in the preceding two paragraphs are based on acute stimulation with IGFs or GLP1 analogs. It is possible that genetic or epigenetic compensation may result from chronic disruption leading to different outcomes from acute manipulation of this pathway. Further investigations will be necessary to reconcile these contrasting findings.

#### Cytoskeleton

An increasing amount of evidence points towards the cytoskeleton as a mechanism by which α-syn causes neurotoxicity in synucleinopathies [130]. A recent paper found that reducing the levels of actin partially ameliorates α-syn-driven locomotor decline and neuronal loss in flies [83]; conversely, increasing the levels of α-spectrin suppressed α-syn-associated neurodegeneration. A bidirectional interaction between α-syn and actin was suspected, when the authors found rod-shaped actin-rich inclusions (actin rods) in fly and mouse brains overexpressing α-syn, as well as postmortem brain samples of synucleinopathy patients. Interestingly, α-spectrin overexpression in fly neurons abolished the formation of actin rods that are associated with α-syn expression, further suggesting an antagonistic relationship between actin and α-spectrin in synucleinopathy.

How do cytoskeletal aberrations affect neurodegeneration? As actin cytoskeleton plays a role in mitochondrial fission via a Drp1-dependent pathway [131–134], Ordonez et al. investigated the relationship between cytoskeleton and mitochondria in α-syn-expressing flies [83]. Their work suggests that α-syn binds to α-spectrin and disrupts its regular subplasmalemmal network, leading to abnormal stabilization of the actin cytoskeleton, which reduces the mitochondrial localization of Drp1, leading to mitochondrial enlargement and dysfunction, subsequently resulting in neuronal death [83] (Figure 4).

Besides the mitochondrial fission-fusion cycle, abnormal actin stabilization by α-syn also impairs autophagy*.* Fly neurons expressing α-syn accumulate aberrantly enlarged autophagosomes [135]. Overexpression of genes encoding actin severing proteins, *GSN* [83,135] *CFL1* [135], reverse abnormal actin stabilization and restore normal autophagy and mitophagy in fly neurons expressing α-syn.

Although reducing *Act5C* levels in fly neurons ameliorates synucleinopathy, *act-5* knockdown in worms is associated with increased α-syn aggregation and decreased mitochondrial content [136]. We also note that α-syn was expressed in muscles in the worm study, while it was expressed in neurons in the fly papers. It is possible the different structural and functional roles of actin in muscles and neurons, rather than species differences, is responsible for the contrasting results with regards to the role of cytoskeleton in synucleinopathy. Additionally, *act-5* is the most divergent of the five actin isoforms in the worm, with a specialized function in the microvilli and does not appear to have a clear ortholog in flies or humans [137,138]. Therefore, unlike *Act5C*, it is not possible to infer a conserved *SNCA* modifier role for *act-5* in PD in humans.

#### α-Syn accumulation

Given that total α-syn level is thought to contribute to the misfolding of α-syn into pathological aggregates, transcriptional upregulation of α-syn would naturally be thought to lead to disease. In rare cases of familial synucleinopathy involving duplication and triplication of the *SNCA* locus, *SNCA* dosage and α-syn levels are correlated with age of onset and disease severity [19,20,30,139,140]. Similarly for sporadic PD, analysis of an intronic SNP in *SNCA* identified it as a cis-regulatory element that binds brain-specific transcription factors, EMX2 and NKX6–1, to reduce *SNCA* transcription [32]. Additional work by others have identified CCAAT/enhancer binding protein δ (C/EBPδ) as a transcriptional repressor of *SNCA* [141], while p53 and GATA-2 are transcriptional activators [142,143]. Furthermore, ZSCAN21 has been identified to activate *SNCA* expression [144,145], but the lack of effect on *SNCA* expression after *ZSCAN21* knockdown in the rat brain suggests that its function may be dependent on the developmental stage of the neurons [146]. As postmitotic neurons live a long time before dying in cases of synucleinopathy, even minor differences in *SNCA* expression levels may have cumulative effects of clinical significance. Hence, further investigations into the transcriptional regulation of *SNCA* is likely to yield more disease-relevant insights.

As neurotoxicity of elevated α-syn largely depends on its misfolding and aggregation, chaperones play important protective roles. It is therefore pertinent that one of the first genetic modifiers of *SNCA* is HSP70; co-overexpression of HSP70 and *SNCA* in fly dopaminergic neurons promoted neuronal survival, while HSP70 and its partner HSP40 were found localized to Lewy bodies in PD brains [147]. Recent NMR characterization revealed that diverse classes of chaperones interact predominantly with α-syn at its N-terminus and the region around tyrosine 39 and prevent aggregation [148]. Interestingly, pharmacological or shRNA inhibition of the chaperones caused relocalization of α-syn to mitochondria, where it presumably forms aggregates. Burmann et al. also found that phosphorylation of tyrosine 39 disrupts chaperone-α-syn interactions, supporting a possible mechanism by which phosphorylation at this residue by the Abelson kinase (ABL1) drives synucleinopathies [149]; we note here that nitration at tyrosine 39 that occurs in response to nitrative insult has also been found to induce aggregation [150,151]. Besides classical chaperones of the HSP70 and HSP90 families, other molecules like DJ-1 (encoded by the PD gene *PARK7*) and synphilin-1 (encoded by *SNCAIP*) have been shown to display chaperone-like activities that reduce α-syn toxicity [152–157].

Defects in protein degradation may also lead to the pathological accumulation of α-syn. Degradation of α-syn may occur via both major protein degradation pathways, the proteasomal degradation pathway and the autophagy-lysosomal pathway (referred to as autophagy henceforth) [158–161]. Within the umbrella of autophagy, α-syn is known to be degraded through either macroautophagy or chaperone-mediated autophagy [162,163]; the latter type of autophagy involves a translocation of unfolded polypeptides into lysosome for degradation, with the help of HSP70. A large volume of research on α-syn degradation and the associated post-translational modifications has been covered by excellent reviews [164–166]. Below, we will highlight a few key findings.

There has been some debate on the relative importance of proteasomal degradation and autophagy on α-syn degradation [163,167]. By performing live brain imaging on transgenic mice expressing GFP-tagged α-syn through cranial windows, Ebrahimi-Fakhari et al. showed that the administration of inhibitors of either proteasomes or autophagy induced accumulation of the α-syn-GFP fusion protein [167]; the authors further showed that normal, endogenous levels of α-syn in non-transgenic mice are maintained by proteasomes, but not autophagy, while elevated levels of α-syn in transgenic mice is prevented from accumulating by a combination of proteasomal degradation and autophagy.

As ubiquitination is a major mechanism by which α-syn is targeted for degradation, ubiquitin ligases have been identified as genetic modifiers of α-syn. For example, NEDD4, a druggable E3 ubiquitin ligase, and its yeast homolog have been found responsible for ubiquitination of α-syn and targeting it for autophagic degradation [168,169]. Two E3 ligases, SIAH1 and SIAH2, monoubiquitinate α-syn to drive it towards either proteasome degradation or aggregation [170–173].

**Figure 5:**
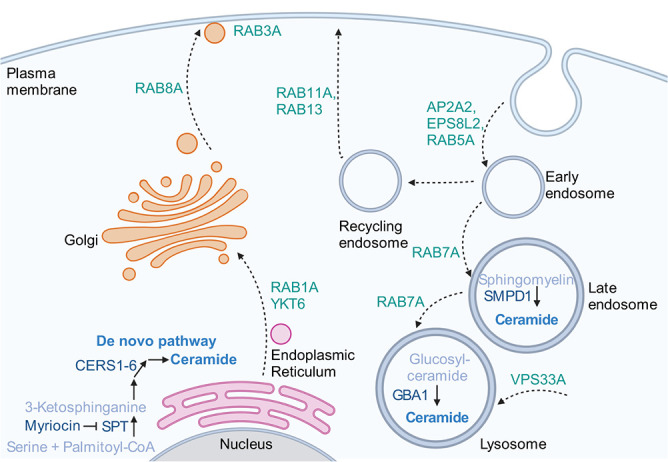
Genetic modifiers of α-syn pathology involved in membrane trafficking (green) ceramide/sphingolipid metabolism (blue). Created with BioRender.com

Besides genes that directly target α-syn for proteasomal degradation and autophagy, additional genetic modifiers have regulatory roles in influencing α-syn degradation. One of the most prominent modifiers of α-syn accumulation is glucocerebrosidase, GBA, one of the most common genetic risk factors of PD [174]. Mutant GBA impairs lysosomal degradation of α-syn by causing aberrant accumulation of sphingolipids in the lysosome (Figure 5) and blocking chaperone-mediated autophagy [175,176]. Consistent with the importance of autophagy in buffering against α-syn toxicity, transcription factor EB (TFEB) regulates the autophagy pathway through the mTOR pathway and promotes the clearance of α-syn toxic oligomers [177].

Other than the generic protein clearance pathways, there is a protease that targets toxic α-syn species. *HTRA2*, encoding a serine protease called Omi, can specifically recognize and precisely degrade toxic α-syn oligomers, but not its monomers thought to be important for normal function at the synapse [24,178–180]. In the fly, Omi expression in all neurons rescued locomotor defects and increased survivability due to α-syn expression, showing its potential as a candidate for therapeutics [178]. Interestingly, Omi was previously identified as a target of phosphorylation by PINK1, which may increase its protease activity [181]. Hence, targeting such proteases with specificity for toxic α-syn species is a viable option for therapeutics.

The importance of manipulating α-syn abundance as a candidate therapeutic strategy cannot be overstated. The above section only provides a glimpse of modifiers in this category and the reader may refer to excellent reviews here [182,183].

#### Membrane trafficking and lipid metabolism

Numerous genes involved in membrane trafficking are genetic modifiers of α-syn (Table 2**;** Figure 5). Proteins with neuroprotective function include Rab1a [184–186], Rab3a and Rab8a [186], which regulate ER-to-Golgi trafficking, post-Golgi trafficking and neurotransmitter tethering and docking, respectively. In addition, AP-2, which is encoded by *AP2A2*, is essential for the formation of endocytic pits and vesicles [187]. On the other hand, the yeast homolog of VPS33A is important in vacuolar biogenesis; its depletion is associated with increased α-syn inclusion formation [188].

Endolysosomal membrane trafficking is known to depend on optimal metabolism of sphingolipids and cholesterols (Figure 5) [189]. In a recent genetic modifier screen that is not yet peer-reviewed, fly homologs of human lysosomal storage disorder (LSD) genes were considered, yielding homologs of 14 human LSD genes that enhanced defects in locomotion of flies expressing α-syn upon knockdown [190]. Among the genes recovered, two cause human cholesterol storage disorders, *LIPA* and *NPC1*, implicating cholesterol accumulation in pathways leading to α-syn toxicity [190]. In a screen performed in worms, the worm homolog of neutral cholesterol ester hydrolase 1 (NCEH1) was found to protect dopaminergic neurons against α-syn toxicity; NCEH1 appears to act via LDLR-mediated cholesterol endocytosis, cholesterol efflux and the synthesis of neurosteroid that lowers mitochondrial ROS [191]. This finding supports a connection between cholesterol metabolism and the mitochondrial dysfunction observed in synucleinopathies. While lower concentrations of cholesterol fed to worms are correlated with lower levels of α-syn-associated dopaminergic neurodegeneration, toxicity due to higher levels of cholesterol supplementation is likely a result of accumulation of cholesterol ester [191]. On the other hand, treatment with statins to inhibit sterol production showed that higher membrane sterol concentrations may be protective in yeast by promoting the binding of α-syn to the plasma membrane [192]. While the yeast study suggests the interesting possibility of using statins as a prophylactic against PD, meta-analyses suggest that the relationship between statin use and PD risk is still controversial [193,194].

Metabolism of ceramides, which include sphingolipids and gangliosides, are implicated in α-syn pathology (Figure 5), perhaps due to their roles in cell signaling and membrane trafficking [189,195]. Ceramide accumulation has been implicated to play a role in α-syn–mediated neurodegeneration [196], while loss of enzymes found upstream in ceramide catabolism pathways, including *GBA1* and *SMPD1*, is described to enhance α-syn pathology [197–199], potentially due to downregulation of the salvage pathway where sphingolipids are recycled to form ceramides that can then be used to produce the various gangliosides needed for proper cell function. Indeed, treatment with exogenous ceramide or inhibition of acid ceramidase to restore ceramide levels reverses defects associated with loss of *GBA1* [198]. Interestingly, selective depletion of subsets of gangliosides by disrupting the gene encoding a ganglioside biosynthetic enzyme, *B4galnt1*, leads to aberrant accumulation of α-syn oligomers, dopaminergic neuron death and motor deficits in mice [200]; furthermore, these phenotypes could be rescued by a brain-permeant analog of GM1 ganglioside. Hence, supplementation of a ceramide species can be neuroprotective in cells with specific deficiencies in related metabolic pathways.

In contrast, reducing the overall level of ceramides may be protective in a PD model. In two related studies, fly models of the *PARK14/PLA2G6* monogenic form of PD show neurodegeneration associated with α-syn aggregation and ceramide accumulation [196,201]. Here, inhibiting *de novo* synthesis of ceramides using myriocin was neuroprotective in flies overexpressing α-syn [196]. Consistent with these data, a subsequent work using human SH-SY5Y cells showed that myriocin alleviated oxidative stress and α-syn inclusions induced by application of α-syn preformed fibrils (PFFs) in the culture medium [202]. However, while myriocin suppressed α-syn toxicity in flies and a human neural cell line, it was instead toxic to yeast cells expressing α-syn [203]. Intriguingly, the yeast synthetic lethality associated with α-syn and myriocin requires only 1 μM myriocin, while SH-SY5Y cells subjected to α-syn PFFs were alleviated α-syn toxicity at 50 μM myriocin. As myriocin possesses powerful antifungal properties [204,205], the synthetic lethality in yeast caused by α-syn and myriocin may be due to the exquisite susceptibility of fungal metabolism to myriocin. Nevertheless, myriocin has shown beneficial effects in non-PD mouse models of neurodegeneration [206,207], suggesting that reduction of ceramide levels might have general neuroprotective effects.

Genetic modifiers may also exert their protective effects by remodeling the lipid membrane, hence influencing the membrane binding propensity of α-syn [208]. As a membrane-driven process, autophagy may also be affected by abnormal cellular lipid profiles, resulting in accumulation of α-syn aggregates due to reduced clearance [209]. In summary, several genes encoding proteins involved in lipid metabolism are implicated in α-syn accumulation due to impaired clearance, leading to greater toxicity.

#### α-Syn spread

**Figure 6:**
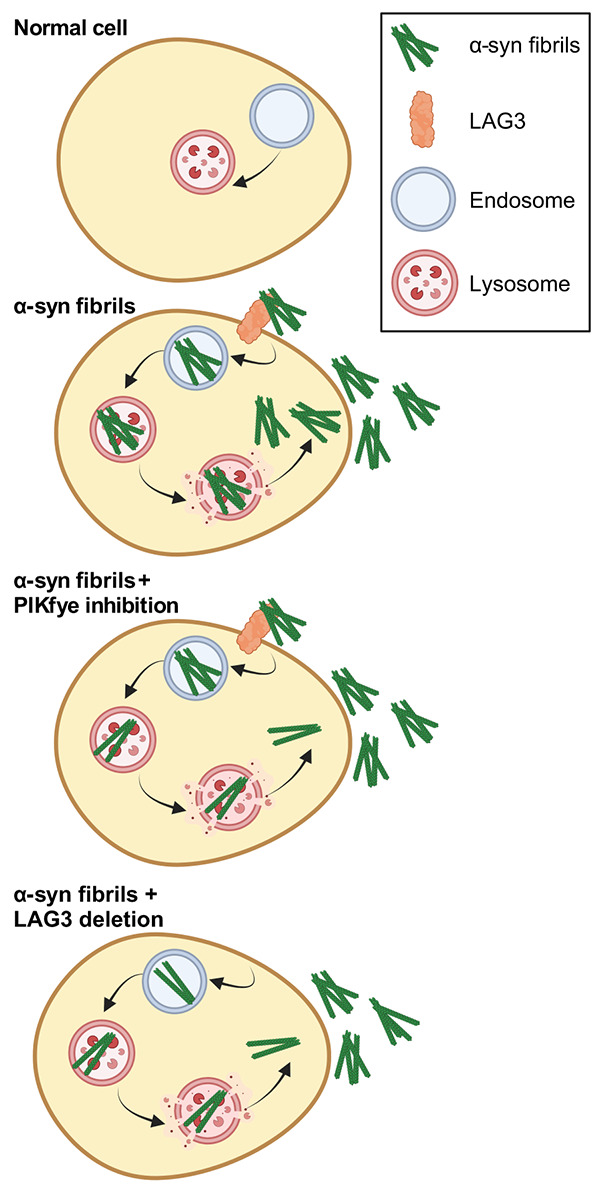
Pathways by which genetic modifiers of α-syn pathology may modulate α-syn spread. Created with BioRender.com

Prion-like spreading of α-syn has been hypothesized and observed *in vitro* in cell culture, and in animal models, as previously reviewed [210–212]. Screens have been conducted to elucidate the pathways involved in α-syn spreading. A useful screening tool, bimolecular fluorescence complementation (BiFC) has been used to track the spread of α-syn aggregation, whereby α-syn is fused to two different fluorescent proteins and their fusion is triggered by addition of α-syn fibrils [213,214]. In an article that has not been peer-reviewed, a genome-wide screen in HEK293T cells was described which identified *PIKFYVE* and the phosphatidylinositol pathway as modifiers of the spread of α-syn aggregation, showing that PIKfyve inhibition prevents α-syn fibrils from reaching the lysosome [213] (Figure 6). Given the involvement of cellular trafficking and autophagy pathways in α-syn spread, genetic and pharmacological modulation of autophagy was predictably found to alter α-syn transmission in worms [214].

Two key steps of α-syn spread are the uptake of synuclein fibrils by the recipient neuron and the subsequent aggregate formation. In a small genetic screen in which neurons exposed to synuclein PFFs were screened with lentiviral knockdown vectors expressing shRNA targeting selected genes, *LRRK2* was found to promote the formation of α-syn aggregates after PFF uptake [215]. Using a different strategy, Mao et al. sought to identify the cell surface receptor responsible for the uptake of PFFs. In this screen from a library of transmembrane proteins in SH-SY5Y cells, LAG3 was identified to preferentially bind PFFs over α-syn monomers (Figure 6) [216]. Upon further characterization, α-syn binding to LAG3 was shown to be required for clathrin-mediated endocytosis of α-syn; the loss of LAG3 rescued neurodegeneration and motor defects in mice injected with α-syn PFF [216]. Hence, this study identifies LAG3 as a candidate therapeutic target for synucleinopathies.

Besides uptake, α-syn spread is also mediated by its secretion. Interestingly, two genetic modifiers of α-syn secretion are the H50Q and G51D mutations in the protein itself [217,218]. Multiple reports indicate that inhibition of autophagy leads to increased α-syn secretion [219–222], hence it is possible that the above two SNCA mutations enhance secretion at least in part through the disruption of autophagy. Another report implicates *ABCC8*, which encodes SUR1, as a genetic modifier of α-syn transfer [223]. Specifically, it was shown that activation of SUR1-K_ATP_ channels leads to hyperpolarized membranes in GABAergic neurons, decreasing GABA release and activation of presynaptic GABA_B_ receptors, reducing the inhibition of Ca^2+^ channels, such that the increase in intracellular Ca^2+^ triggers α-syn release from the neuron [223]. Additional studies have implicated tubulin polymerization-promoting protein (*TPPP/p25α*) and *ATP13A3*/*PARK9* as modifiers of α-syn secretion [224,225].

As α-syn spreading is a topic of intense research, we expect a better understanding of this process in the near future. Hopefully, this would translate into further therapeutic strategies for synucleinopathies.

#### Neuroinflammatory responses

**Figure 7:**
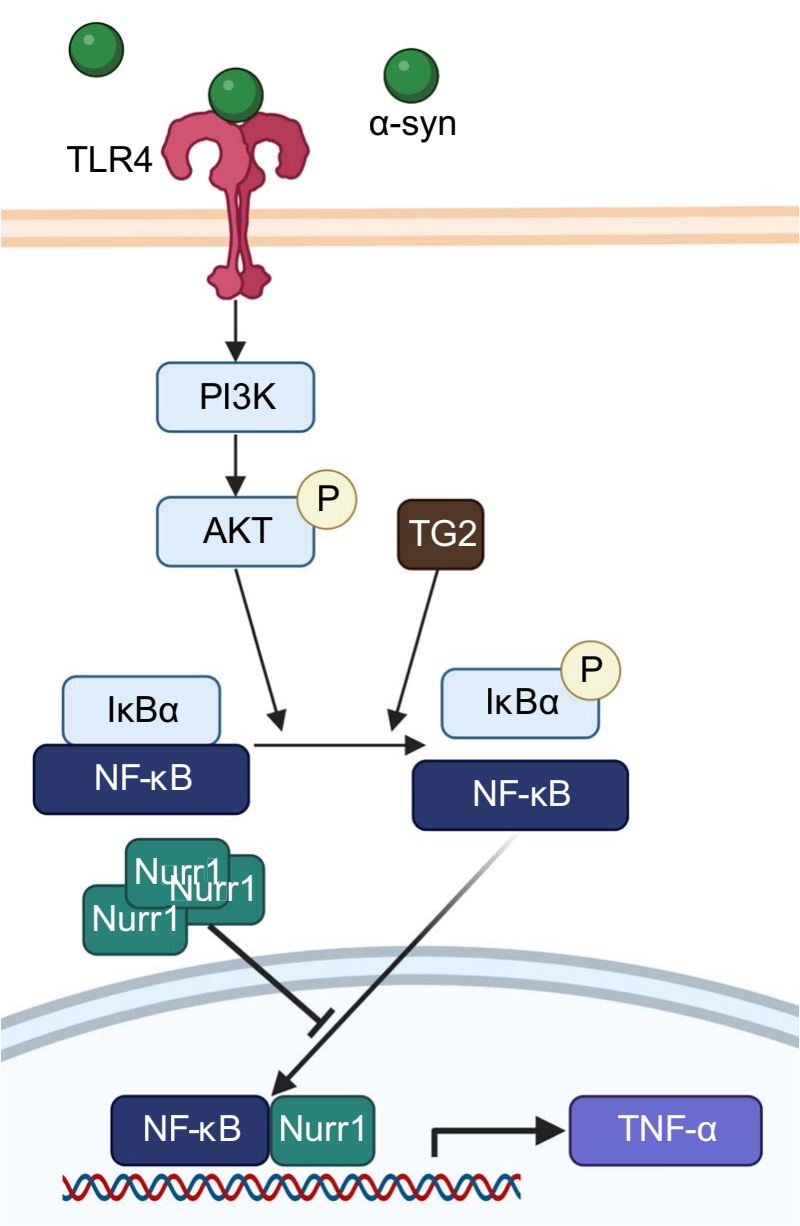
Pathways by which genetic modifiers of α-syn pathology may lead to neuroinflammatory responses such as the production of cytokine TNF-α. created with BioRender.Com

Besides neurons, the spread of extracellular α-syn affects microglia, the resident innate immune cells of the brain and astrocytes, hence triggering neuroinflammatory responses [226–228]. A number of genes involved in neuroinflammatory responses have been identified as genetic modifiers of α-syn, mainly from the TLR4/NF-κB signaling pathway. Deletion of *TGM2*, encoding the transglutaminase 2, TG2 which is involved in NF-κB-mediated inflammation [229,230], attenuates neuroinflammatory responses to α-syn in transgenic mice [231]. A different work showed that Nurr1 overexpression inhibited nuclear translocation of NF-κB, and partially attenuated the increased production of cytokine TNF-α induced by α-syn [232] (Figure 7). Besides this, Rev-erbα, encoded by *NR1D1* that was recovered from our screen [48], was also recently shown to attenuate neuroinflammation in an MPTP-induced mice PD model, likely by reducing NLRP3 inflammasome activation by NF-κB [233].

### *LRRK2* and the sirtuins—pleiotropic modifiers of *SNCA*

*LRRK2* mutations are the most common genetic causes of PD [174]. It encodes a large ~280 kD protein with two enzymatic domains—a Ras of complex (ROC) GTPase domain and a serine/threonine kinase domain [234]. It has been implicated in a wide range of cellular functions, based on its kinase substrates that include proteins involved in synaptic vesicle endocytosis, microtubule network, protein translation and several members of the Rab GTPases that function in various cellular processes [234]. Brain RNA Seq data indicate that *LRRK2* is expressed mainly in microglia and oligodendrocytes, with lower levels of expression in neurons and astrocytes [23,235]. Knockin mice expressing the pathogenic R1441G mutant protein from the endogenous promoter accumulates increased levels of intracellular and extracellular α-syn, indicating that *LRRK2* is a *SNCA* modifier. Although much of *LRRK2* is expressed outside neurons, many studies examined neuron overexpression of gain-of-function mutations of *LRRK2*. For example, mouse brains co-expressing the G2019S *LRRK2* mutant with the A53T *SNCA* mutant showed accumulation of high molecular weight species of α-syn, enhanced striatal neuron loss, synergistic disruption of Golgi network, microtubule network and mitochondrial functions [236]. Similarly, co-expression of *LRRK2* mutants and SNCA in fly neurons enhanced neurodegeneration [237]. A difference between the fly and mouse studies was that human A53T SNCA expression combined with *LRRK2* loss-of-function mutation in mouse suppressed high molecular weight α-syn accumulation and neuron loss, while *LRRK2* loss-of-function in *SNCA*-expressing flies enhanced neurodegeneration phenotypes [236,237]; it is possible that some of the pleiotropic functions of *LRRK2* may have diverged between flies and mice. Finally, the PD risk gene *LRRK2* is found to be highly associated with microglia in the single-nuclei transcriptome of sporadic PD midbrain, suggesting a role in neuroinflammation [238]. Indeed, upon exposure to α-syn PFFs, primary microglia cells with a *LRRK2* loss-of-function mutation shows attenuated activation compared to controls [239], while gain-of-function mutations in mice injected with PFFs display increased microglia activation. Hence, this finding suggests that LRRK2 may be a drug target for mitigating neuroinflammation in synucleinopathies.

The sirtuins are a family of genes encoding NAD+-dependent histone deacetylases consisting of *SIRT1–7* in humans, each implicated with diverse functions. For this review, we will discuss only SIRT1, 2 and 3, focusing on the characterization of these 3 genes in PD models. While SIRT1 is localized to the cytoplasm in the adult mouse brain, SIRT2 is known to undergo nuclear-cytoplasmic shuttling and SIRT3 is localized to mitochondria [240,241]. Studies of sirtuins in a variety of experimental models have suggested pro-aging or anti-aging, as well as, pro-neurodegeneration or neuroprotective functions in various contexts [240,242].

SIRT1 has been shown to be neuroprotective in mammalian models of synucleinopathy. Using viral overexpression of *SNCA* in mouse primary neurons and a mouse dopaminergic cell line, SIRT1 activation by a selective agonist, SRT2104, was shown to reduce cell death; this neuroprotective effect of SRT2104 was attributable to SIRT1 as it could be reversed by a SIRT1 inhibitor, EX527 [243]. Additional investigation suggests that SIRT1 activation was associated with restoration of mitochondrial function and autophagy that were impaired by *SNCA* overexpression. Specifically, pharmacological activation of SIRT1 was associated with increased nuclear localization of TFEB, a transcription factor that upregulates autophagy, and various markers of autophagy flux. Another group reported that activation of SIRT1 by resveratrol reduced dopaminergic neuron loss and restored locomotor functions in mice injected with MPTP in the substantia nigra; the authors attributed the neuroprotective action of resveratrol to SIRT1 by reversing them with the SIRT1 inhibitor, EX527 [244]. Interestingly, they found that the above SIRT1 activation correlated with deacetylation of the autophagy protein LC3 in the striatum, where axons from substantia nigra terminate; this suggests that LC3 may a direct target of the deacetylase activity of SIRT1.

Unlike SIRT1, SIRT2 has been shown to be pro-neurodegeneration in PD models. A chemogenetic study identified a series of small molecule inhibitors of SIRT2 that specifically inhibit SIRT2, but do not affect SIRT3 activities [245]. These inhibitors were shown to suppress α-syn toxicity in human H4 neuroglioma cell line and cultured rat midbrain primary neurons that were transfected with *SNCA*; they also suppressed dopaminergic neuron loss in transgenic *Drosophila* overexpressing *SNCA*. A follow-up study by the Outeiro group suggests that SIRT2 may promote α-syn toxicity, at least in part, through the removal of acetyl groups from lysine residues on α-syn [246]. They showed that endogenous α-syn is acetylated and can be deacetylated by SIRT2. They further compared two artificial α-syn mutations at K6 and K10 that either mimic acetylation or the non-acetylated states; the acetylation mimic version was less toxic than the acetylation-resistant one when overexpressed in cultured rat primary neurons and in rat substantia nigra using viral vectors. Taken together, these findings suggest that deacetylation of α-syn at lysine residues by SIRT2 may promote neurodegeneration.

The mitochondrial localized SIRT3 has been shown to be neuroprotective. In rats that were unilaterally injected with viral vectors in the substantia nigra, co-expression of SIRT3 with *SNCA* partially suppressed dopaminergic neuron loss and locomotor defects [247]; this neuroprotective function depends on the deacetylase activity as the catalytic mutant of SIRT3 was unable to rescue the α-syn toxicity. Consistent with this finding, SIRT3 protein levels are downregulated in post mortem brains with Lewy body disease and rat brains overexpressing α-syn [248].

Besides studies performed in mammalian systems, sirtuins have been found to be *SNCA* modifiers in worms and yeast. A large unbiased screen in worms using overexpression of GFP-tagged α-syn in muscles identified *sir-2.1*, whereby disruption of this gene increased the number of α-syn inclusions [249]. In contrast, a yeast study found that a candidate *SIRT1* ortholog, *sir2*, is essential for *SNCA* toxicity and deletion of this gene suppressed autophagy and rescued the yeast cells [250]. While both the worm *sir-2.1* gene and the yeast *sir2* gene are candidate orthologs of *SIRT1*, it seems that one has neuroprotective function while the other is pro-neurodegeneration. Given that there are 4 worm sirtuins and 5 yeast sirtuins compared to 7 human counterparts, one must use caution when inferring orthology between human sirtuins and their yeast or worm counterparts, due to the relatively low levels of sequence conservation. In addition, it is intriguing that in yeast study on *sir2*, suppression of excessive autophagy was protective against cell death, in contrast to the findings on SIRT1 and others in the preceding section on α-syn accumulation. It is possible that yeast cells are less equipped than neuronal cells to high levels of α-syn, given that the latter are adapted to high endogenous levels of this protein [29] and are able to upregulate autophagy for degrading excess α-syn [167].

#### Other pathways

Pathological α-syn aggregates have shown to disrupt calcium (Ca^2+^) homeostasis [251]. This may be through its modulation of voltage-gated Ca^2+^ channels, intracellular Ca^2+^ channels, formation of Ca^2+^ permeable pores, disruption in lipid membrane composition and packing, even ER or mitochondrial dysfunction [252]. As mentioned above, calcium overload due to α-syn overexpression leads to dysfunctions in mitochondria and autophagy, resulting in cell death, likely by acting through TLK2 [253]. Besides this, calcium was reported to bind and activate calcineurin and calmodulin, which dephosphorylates NFATC4, which then localizes to the nucleus to activate a toxic program [254]. However, both deletion and overexpression of calcineurin were found to exacerbate α-syn toxicity in yeast [254]. Calcineurin likely confers its protective effects through cAMP-responsive element-binding protein-regulated transcription coactivator 2 (CRTC2) [255], which can be achieved by limiting but not eliminating the availability of calmodulin, calcineurin and their substrates [254]. Hence, Ca^2+^ homeostasis and downstream pathways including that involving calcineurin are also important in synucleinopathies.

Dysregulation of the cell cycle has been demonstrated in synucleinopathies and in PD models [256–259]. The human orthologs of two genes from our *SNCA* modifier screen have been implicated in this pathway elsewhere (*CDC27*, *HECA*) [48,129,260,261], perhaps implicating its relevance to cause disease in synucleinopathies. While few other genetic modifiers of α-syn seem to be involved in this pathway (Table 2), *PARK7* was also recently found to cause aberrant cell cycle re-entry, thus resulting in neuronal death [262].

Another pathway poorly represented in our list is circadian rhythm (Table 2), despite being one of the most common non-motor symptoms of PD [263] and associated with PD risk [264], even suggested to cause neurodegenerative diseases [265,266]. One of the fly *SNCA* modifiers from our screen, *Eip75b*, is homologous to *NR1D1* and *NR1D2*, which are core components of the circadian machinery and are involved in dopaminergic regulation [61,267]. In addition, *Drosophila MTA1-like* which is found in our *SNCA* modifier screen [48], has two human orthologs which are implicated with circadian rhythm-related functions: *MTA1*, which is an integral component of the circadian transcriptional circuit [268] and *MTA3*, which is associated with insomnia in a large GWAS meta-analysis [269].

## *SNCA* MODIFIERS AND PATHWAYS TARGETED IN PD CLINICAL TRIALS

How might the discovery of *SNCA* modifiers help in the development of therapeutic interventions? An analysis of previous drug development pipelines estimates that drugs with targets that are supported by human genetics evidence are twice as likely as those with none to progress from Phase I clinical trials to approval [270]. For example, the discovery of *PCSK9* has led to the development and approval of Inclisiran for hypercholesterolemia [271]. Inspired by such findings, a few biotech startups now aim to screen genetic modifiers for druggable pathways [272–274]. While no disease-modifying interventions have been approved for PD, several candidates are currently in clinical trials. In this section, we shall discuss selected clinical trials in light of the above findings on *SNCA* modifiers (Table 3), with a focus on PD where an increasing number of interventions are emerging in the pipeline.

**Table 3:** PD clinical trials of disease-modifying interventions

Drug	Drug action	Main outcome	Phase of clinical trial	References	Year(s) of publication
Exenatide	Glp-1 receptor agonist	Positive effects on motor scores.	2	[278–280]	2014, 2017, 2019
Pioglitazone	PPARα & PPARγ agonist	Did not modify progression in early PD.	2	[282]	2015
GM1 ganglioside	Modulator of sphingolipid metabolism	Reduced worsening of motor symptoms.	Single center RCT	[285–287]	2010, 2013, 2015
Recombinant GBA1	Catabolism of glucosylceramide	GBA was permeable through BBB. Demonstrated safety.	1	[288]	2022
Venglustat	Glucosylceramide synthase inhibitor	Failed to provide benefit for motor symptoms (Sanofi press release).	2	[289–291]	2021
Prasinezumab	Anti-aggregated α-syn antibody	No difference from placebo in motor scores and in dopamine transporter levels in putamen.	2	[292]	2022
UCB0599	Inhibitor of α-syn misfolding	Demonstrated safety.	1	[532]	2022
Anle138b	Inhibitor of α-syn misfolding	Demonstrated safety.	1	[533]	2022
Deferiprone	Iron chelator	Motor symptoms worsened despite reduction of iron in SNc.	2	[295]	2022
Nilotinib	ABL kinase inhibitor	Biomarker engagement data conflicting between 2 studies. Motor symptoms showed negative trend in Simuni et al. (2021)	2	[534,535]	2020, 2021
Intranasal glutathione	Antioxidant	No symptomatic benefit compared to placebo.	2b	[296,297]	2015, 2017
Isradipine	L-type calcium channel blocker	Did not slow progression of early PD.	2	[298,536]	2013, 2020
Nicotinamide Riboside (NR)	Precursor to NAD, a cofactor of sirtuins	Oral NR therapy increases brain NAD levels and impacts cerebral metabolism in PD.	1	[537]	2022
Infusion of GDNF into putamen	Neuroprotective trophic factor	No motor improvement over placebo, but 18F-DOPA uptake increased in putamen.	Single center RCT	[299–301]	2006, 2007, 2019

RCT, randomized control trial

### Insulin signaling

One candidate that has shown promising clinical data is exenatide, a glucagon-like peptide 1 (GLP1) analog, which is currently used for treating type 2 diabetes. Endogenous GLP1 is known to augment insulin signaling and have neuroprotective functions [126,275]; the likely mode of action of GLP1 and its analogs is by enhancing insulin sensitivity through the action of SirT1 on IRS [118,124,125]. As PD has been associated with brain biomarkers of insulin resistance in humans [276,277], it is thought that relieving brain insulin resistance may ameliorate the progression of PD pathology. A single-center Phase 2 trial of exenatide on PD showed improvement of motor scores that was sustained beyond the period of exposure [278,279]. Furthermore, analysis of neuron-derived exosomes from subjects in this trial revealed exenatide treatment had augmented tyrosine phosphorylation of IRS1, suggesting that activation of neuronal insulin signaling by exenatide is correlated with locomotor benefits [280]. Consistent with the above PD trial, GLP1 analogs have been found to benefit dementia patients in another clinical study [281]. Hence, GLP1 analogs are likely to provide a protective function against synucleinopathy and perhaps neurodegeneration, in general, by alleviating brain insulin resistance.

While exenatide has shown promising data in the above study, another diabetes drug, pioglitazone, did not modify progression in early PD in a different Phase 2 trial published in 2015 [282]. The mechanism of action of pioglitazone differs from that of exenatide in that the former is an agonist of peroxisome proliferator-activated receptors (PPARs) PPARα and PPARγ [283]. Currently, it is not clear whether the difference in efficacies against PD between pioglitazone and exenatide is due to the two drugs targeting different aspects of insulin signaling or other less obvious reasons.

Notwithstanding negative results from the pioglitazone trial, promising data from the exenatide studies suggests that enhancing the insulin/IGF signaling pathway in the brain is a viable target. This is consistent with the identification of IGF1 and IGF2 as *SNCA* modifiers that ameliorate α-syn accumulation and cytotoxicity in cell lines and a 6-OHDA mouse model of PD [120,121]. While there are contrasting data from worm and fly models of synucleinopathy showing that reduced insulin signaling is neuroprotective [106], it is possible that different PD models may reflect different stages of synucleinopathy that may or may not match those of the PD patients who participated in the clinical trials.

### Ceramide metabolism

Ceramides, sphingolipids and gangliosides play important roles in cellular signaling and membrane trafficking [189,195]. Based on the observation that GM1 ganglioside level is reduced in PD patients [200,284], Jay Schneider and colleagues conducted a randomized control trial and showed that supplementation of GM1 ganglioside can delay the progression of motor symptoms in PD [285,286]. Positron emission tomography imaging of dopamine transporter binding by a tracer suggests that GM1-treated subjects showed slower loss of striatal dopaminergic synapses, suggesting a disease-modifying effect of GM1 on PD [287].

Two other drug trials that target ceramide metabolism involve putaminal recombinant GBA1 delivery and Venglustat. The GBA1 Phase 1 trial demonstrated that a sophisticated ultrasound technique is able to deliver GBA1 across the blood–brain barrier safely with signs of target engagement [288]. Venglustat is a glucosylceramide synthase inhibitor developed by Sanofi to alleviate the accumulation of glycosphingolipids, including glucosylceramide, which is thought to impair lysosome function. The Venglustat trial for PD has failed to demonstrate benefits to motor function even though glucosylceramide levels decreased in cerebrospinal fluids, indicating successful target engagement [289–291].

The preliminary success of the PD trials with GM1 ganglioside and the failure of Venglustat suggest that further understanding of ceramide metabolism and its role in brain physiology is needed. Nevertheless, this pathway appears to provide a fruitful avenue for more investigations on potential PD interventions.

### Other pathways

Table 3 lists additional trials of candidate disease-modifying treatments, including those that investigate drug safety profiles and those that tested efficacies. The following are some trials that have failed to demonstrate efficacy.

*Anti-aggregated α-syn antibody, Prasinezumab*: This trial aimed to prevent the spread of aggregated α-syn. However, the motor scores and dopamine transporter levels in the putamen were not different from placebo [292].

*Iron chelator deferiprone*: Potential mechanisms of action—ameliorate iron over-accumulation [293] or activate HIF1A [294]. Motor symptoms worsened despite reduction of iron in SNc [295]; possibly due to depletion of the iron co-factor from tyrosine hydroxylase.

In addition, the following PD trials also failed to demonstrate efficacy: intranasal glutathione delivery, intraputaminal delivery of GDNF and Isradipine, an L-type calcium channel blocker [296–301].

While disappointing, it is worth investigating how each trial might have failed (or succeeded) to demonstrate efficacy. One could ask whether target engagement was successful. Was the hypothesis that guided the intervention correct? Was the stage of PD at which the intervention was administered allow for the hypothesized mechanism to occur? Could the pleiotropic nature of a drug, such as deferiprone, have resulted in negative actions outweighing positive ones?

While experimental models of PD have allowed researchers to gain many mechanistic insights, there are many aspects of synucleinopathies that are not captured by models. Hence, it is important to go beyond clinical outcomes in these trials to investigate underlying mechanisms of the interventions, using biomarkers and brain imaging methods.

## FUTURE PERSPECTIVES

### Challenges in modeling synucleinopathies

In trying to model progressive neurodegeneration with complex etiologies, experimental models of synucleinopathies face several challenges. First, it is a challenge to model the effect of aging on synucleinopathy; disease progression occurs in time scales of decades in humans, while experimental models like yeast, worms, flies and mice have lifespans ranging from days to three years. When modeling synucleinopathy in cultured cells or organoids, it is unclear what *in vitro* correlate of biological aging should be [302]; in cases where proliferating cells are used to model post-mitotic neurons, it is possible that differences in cellular physiology may be a confounding factor [303]. Second, in attempting to accelerate neurodegeneration in models, experimenters may be applying genetic or chemical insults that are more intense than what occurs in humans. It is not clear whether biological responses to long-term mild insults are different from those to acute strong insults. Third, species differences between human and model organisms may be yet another confounding factor [304]. Although organisms with nervous systems possess sophisticated cell–cell interactions in their brains, that could still be challenging to model using human-derived neurons [305].

Notwithstanding the above limitations, the identification of *SNCA* modifiers in model organisms that are homologous to genes underlying monogenic forms of PD and sporadic PD suggests that substantial genetic contributions in synucleinopathies are evolutionarily conserved [48,306–308]. The diverse characteristics of experimental models may allow different but complementary sets of genes to be discovered, thus expanding our knowledge of synucleinopathies.

### A multi-step hypothesis of PD progression

The increase of PD incidence with age has prompted researchers to propose a multi-hit hypothesis that is analogous to that for cancer progression [309–311]. Using robust, nationwide PD incidence data from New Zealand, Le Heron et al. found that the way PD incidence varies with age is consistent with a multi-step disease progression hypothesis; six steps and eight steps for early-onset and late-onset PD, respectively [309]. In experiments done on rodents, sequential application of lipopolysaccharides or a combination of paraquat and maneb have led to progressive nigral dopaminergic neuron loss in rodents after cessation of toxin exposure [312,313]; this is despite the fact that acute toxin applications typically do not yield progressive degeneration [310].

#### How do SNCA modifiers fit into a multi-step PD progression model?

If the progression of synucleinopathies involves multiple steps, how do genetic modifiers contribute to this process? For sporadic synucleinopathies, it is possible that individual patients may carry multiple variants, each with small effect sizes, which contribute to disease risk. Genes near some PD GWAS have been identified independently or verified in experimental models as *SNCA* modifiers, such as *GBA*, *LRRK2*, *MED13*, *CDC27*, [48,175,314]**.** However, many other *SNCA* modifiers discussed in this review do not have human orthologs lying close to genetic variants associated with PD or DLB. Perhaps, the identification of more GWAS loci would show if more *SNCA* modifiers are indeed genetic predisposition factors. Alternatively, epigenetic modifications instead of genetic mutations at *SNCA* modifiers could contribute to disease progression. In this scenario, it is possible that an environmental insult could contribute to disease progression through the epigenetic modification of an *SNCA* modifier. Here, we take epigenetic changes to broadly mean any mechanism from chromatin modifications to transcriptomic changes to metabolic reprogramming. Hence, it is conceivable that a genetic mutation or an epigenetic change to an *SNCA* modifier could contribute to disease progression.

How could one test whether genetic modifiers of *SNCA* act as sequential hits in a multi-hit experimental model? For example, one could have an experimental model in which one *SNCA* modifier is constitutively mutated while a second modifier is conditionally knocked down in a temporally controlled manner. Such experiments may help establish a sequence of events that may be theoretically possible during the progression of synucleinopathy.

How would one investigate whether there is a sequence of gene actions that contribute to progression in humans? If resources are not limited, one way to address this is to investigate epigenetic changes associated with different brain cell types and brain regions from postmortem samples at different stages of disease progression, as defined by abundance and distribution of Lewy pathology [28,315]. One could then ask whether a group of *SNCA* modifiers consistently undergoes epigenetic changes in the same sequence across samples. Given the heterogeneity of synucleinopathies [316,317], such an approach may even be used to determine whether distinct sequences of epigenetic changes occur in different forms or subtypes of synucleinopathies. Currently, there are many efforts to characterize chromatin modifications or transcriptomic changes in synucleinopathies using bulk tissue or single-nuclei approaches [238,318–321]. Interestingly, single-nuclei transcriptomics create opportunities to computationally reconstruct gene expression changes in specific cell populations, as performed recently on PD brains [238]. Similar strategies may guide efforts to place particular *SNCA* modifiers to specific stages (and cell types) during disease progression.

#### How would a multi-step model guide design of therapeutic interventions?

As synucleinopathies like PD progress in humans, different symptoms emerge that likely reflect changes in the underlying cellular processes. For example, postmortem data show that dopaminergic synapses in striatum suffer larger losses compared to the corresponding cell bodies in substantia nigra, suggesting that neuronal pathology occurs in a synapse-to-soma direction [322]. This suggests that efforts to save the synaptic terminals need to be applied at a relatively early stage of the disease. Similarly, a better knowledge of how synucleinopathy spreads across brain regions correlates with clinical symptoms will allow investigators to infer stage of disease progression of each patient. Being able to place *SNCA* modifiers at different stages of disease progression may allow the identification of druggable targets that are appropriate to specific clinical stages of the diseases. Hopefully, such knowledge would guide investigators towards successful clinical trials for disease-modifying interventions.

## Supplementary Material

Web_Material_kvad001

## Data Availability

All data mentioned in this article has been published elsewhere as referenced.
