# Supplementary material for: Genetic modifiers of synucleinopathies—lessons from experimental models
Source: Oxf Open Neurosci. 2023 Mar 9;2:kvad001. doi: 10.1093/oons/kvad001 (PMC10913850; doi:10.1093/oons/kvad001)
Supplement: Web_Material_kvad001 [file Web_Material_kvad001.zip › Reviewers comments - Supplementary Material - OXFNSC-2022-019.R1 .docx]

**REVIEWER COMMENTS**

**OXFNSC-2022-019.R1 - "Genetic Modifiers of Synucleinopathies - Lessons from Experimental Models"**

**Original submission - OXFNSC-2022-019**

**Reviewer 1**

Comments to the Author

This manuscript reviews a lot of current and past work on the function and modifiers of the SNCA gene. It is very well written and would act as a very comprehensive and useful review to the field. The authors take pains to detail multiple pathways and mechanisms through which alpha-synuclein could be functioning in multiple systems. I have no comments to add to this very well written review and think that it can be published as is.

**Reviewer 2**

Comments to the Author

In this assay the authors discuss multiple genetic modifiers of synucleinopathies identified in studies of various model organisms. The authors describe many pathways and cellular processes involved or affected by aggregation of α-synuclein, with a particular focus on genes that may serve as drug targets. Whereas this review is comprehensive in a sense that it highlights a variety of studies, there are several caveats in the way they describe each modifier. The major point in my view is that for each modifier (e.g., SIRT1, actin, myriocin, LRRK2 etc.) they highlight the effect, enhancement or suppression, on α-syn toxicity, while disregarding the differences between the distinct models. The readers are therefore left with allegedly contradicting roles of the same modifiers without providing satisfying explanations except for stating that this is due to tissue specificity or different organisms. Deeper explanations based on the particularities of each model (regardless of the organism or tissue), as well as description of the similarities and differences among the different models shall be offered, as not all models are equally strong.

Minor points

1. Overrepresentation of the author’s own results (Ren et al. 2022).

2. Page 3 lines 12-19: the sentences are somewhat redundant. Rewrite this paragraph such that the Braak’s hypothesis is not stated twice.

**Decision letter - OXFNSC-2022-019**

16-Jan-2023

Dear Dr. Koh,

Manuscript ID OXFNSC-2022-019 entitled "Genetic Modifiers of Synucleinopathies - Lessons from Experimental Models" which you submitted to the Oxford Open Neuroscience, has been reviewed. The comments of the reviewer(s) are included at the bottom of this letter.

Please note that this journal operates with transparent peer review. This means that if your submission is accepted for publication, the full peer review history of your article will publish online alongside your article. This includes reviewer comments, editor decision letters, and your author responses.

The reviewer(s) have recommended publication, but also suggest some major revisions to your manuscript. Therefore, I invite you to respond to the reviewer(s)' comments and revise your manuscript.

To revise your manuscript, log into https://mc.manuscriptcentral.com/oxfnsc and enter your Author Centre, where you will find your manuscript title listed under "Manuscripts with Decisions." Under "Actions," click on "Create a Revision." Your manuscript number has been appended to denote a revision.

You may also click the below link to start the revision process (or continue the process if you have already started your revision) for your manuscript. If you use the below link you will not be required to login to ScholarOne Manuscripts.

*** PLEASE NOTE: This is a two-step process. After clicking on the link, you will be directed to a webpage to confirm. ***

https://mc.manuscriptcentral.com/oxfnsc?URL_MASK=ba61fd6f8a7047f8af8f2a68b97e3777

You will be unable to make your revisions on the originally submitted version of the manuscript. Instead, revise your manuscript using a word processing program and save it on your computer. Please also highlight the changes to your manuscript within the document by using the track changes mode in MS Word or by using bold or colored text.

Once the revised manuscript is prepared, you can upload it and submit it through your Author Centre.

When submitting your revised manuscript, you will be able to respond to the comments made by the reviewer(s) in the space provided. You can use this space to document any changes you make to the original manuscript. In order to expedite the processing of the revised manuscript, please be as specific as possible in your response to the reviewer(s).

IMPORTANT: Your original files are available to you when you upload your revised manuscript. Please delete any redundant files before completing the submission.

Because we are trying to facilitate timely publication of manuscripts submitted to the Oxford Open Neuroscience, your revised manuscript should be uploaded as soon as possible. If it is not possible for you to submit your revision in a reasonable amount of time, we may have to consider your paper as a new submission.

Once again, thank you for submitting your manuscript to the Oxford Open Neuroscience and I look forward to receiving your revision.

Sincerely,

Senior Editor, Oxford Open Neuroscience

Reviewer: 1

Comments to the Author

This manuscript reviews a lot of current and past work on the function and modifiers of the SNCA gene. It is very well written and would act as a very comprehensive and useful review to the field. The authors take pains to detail multiple pathways and mechanisms through which alpha-synuclein could be functioning in multiple systems. I have no comments to add to this very well written review and think that it can be published as is.

Reviewer: 2

Comments to the Author

In this assay the authors discuss multiple genetic modifiers of synucleinopathies identified in studies of various model organisms. The authors describe many pathways and cellular processes involved or affected by aggregation of α-synuclein, with a particular focus on genes that may serve as drug targets. Whereas this review is comprehensive in a sense that it highlights a variety of studies, there are several caveats in the way they describe each modifier. The major point in my view is that for each modifier (e.g., SIRT1, actin, myriocin, LRRK2 etc.) they highlight the effect, enhancement or suppression, on α-syn toxicity, while disregarding the differences between the distinct models. The readers are therefore left with allegedly contradicting roles of the same modifiers without providing satisfying explanations except for stating that this is due to tissue specificity or different organisms. Deeper explanations based on the particularities of each model (regardless of the organism or tissue), as well as description of the similarities and differences among the different models shall be offered, as not all models are equally strong.

Minor points

1. Overrepresentation of the author’s own results (Ren et al. 2022).

2. Page 3 lines 12-19: the sentences are somewhat redundant. Rewrite this paragraph such that the Braak’s hypothesis is not stated twice.

Associate Editor

Comments to the Author:

Please note the detailed comments by one of the reviewers and revise your manuscript accordingly.

**Author response**

Dear Professor,

Thank you for giving us time to revise our manuscript. We agree with Reviewer 2 that for certain SNCA modifiers, we have not adequately discussed the differences in data between experimental models. In the revised version, have made substantial revision to discuss caveats in each model where there are such differences. Where possible, we have offered our opinion on which set of data provided stronger evidence between differing results. The segments we have amended to discuss in details are:

1. Pages 5-7 - In the section on Mitochondrial functions, we discussed the different changes to mitochondrial morphology in response to synuclein overexpression in different models and the roles of the fission and fusion machinery. We have also amended the paragraph on Miro and mitophagy. Now the paragraph on mitogenesis comes after mitochondrial fission/fusion and mitophagy.

2. Page 12 - In the section on Cytoskeleton, we have added discussion on the contrasting roles of fly Act5C and worm act-5 as modifiers of SNCA in opposite directions.

3. Page 15-16 – In the section on Membrane trafficking and lipid metabolism, we have added sentences to compare the effect of statins on a yeast SNCA model and human epidemiological studies on the relationship between statins and PD risk.

4. Page 16 – In the same section, we discussed the contrasting effects of myriocin on SNCA-expressing flies, human SH-SY5Y cells and yeast.

5. Page 20-22 – We noted that LRRK2 and sirtuins have been discussed in a touch-and-go fashion at different places in the original manuscript. We have decided to consolidate discussions on these two groups of pleiotropic modifiers into a new section. This also allows us to address any difference between findings from different experimental models.

Regarding minor points made by Reviewer 2, we have also made an amendment to remove the redundancy in the sentences concerning Braak’s hypothesis. Regarding the overrepresentation of our own results (Ren et al., 2022), we note that our motivation of writing this review is to place our own findings in the context of numerous earlier studies on SNCA modifiers. Hence, we feel that the 2 paragraphs summarizing our own findings serve as a segue to lead to the large volume of work on these modifiers.

We have used the track changes function on Microsoft Word to highlight the changes we have made. I hope that we have sufficiently addressed the reviewer’s concerns.

Best regards,

Tong-Wey Koh

**Decision letter - OXFNSC-2022-019.R1**

19-Feb-2023

Dear Dr. Koh,

It is a pleasure to accept your revised manuscript entitled "Genetic Modifiers of Synucleinopathies - Lessons from Experimental Models" in its current form for publication in the Oxford Open Neuroscience. The comments of the reviewer(s) who reviewed your manuscript are included at the foot of this letter.

Please note that this journal operates with transparent peer review. This means that the full peer review history of your article will publish online alongside your article. This includes reviewer comments, editor decision letters, and your author responses.

Next steps

You will receive an email from no-reply@scipris.com within roughly one week. This is your invitation to sign up for an account with SciPris, Oxford University Press’ author portal hosted by Aptara. You will need to create an account if you do not already hold one. Please register or log into your account and follow the online instructions which will guide you through signing your licence and paying the APC. The email and the portal have clearly signposted support options if you need any help during this process.

Please note that SciPris is a completely different system from ScholarOne, so your credentials to submit your manuscript here will not work there. Once you’ve created a SciPris account, you will be able to use it whenever you publish with Oxford Open Neuroscience or any OUP journal. Please note that OUP will only ever request payment for applicable fees be made via SciPris or to an OUP bank account. If you ever have concerns about the legitimacy of a request, please do not hesitate to contact a customer services agent via the SciPris portal or directly via oupsupport@scipris.com.

Thank you for your fine contribution. On behalf of the Editors of the Oxford Open Neuroscience, we look forward to your continued contributions to the Journal.

Sincerely,

Senior Editor, Oxford Open Neuroscience

Reviewer: 1

Comments to the Author

I have no further comments. I feel that the manuscript should be published in the current form.

Reviewer: 2

Comments to the Author

The authors addressed most of my comments. I have no further reservations.

Associate Editor

Comments to the Author:

The reviewers found that the authors addressed most of the comments.
